# Development and Evaluation of an Ergonomically Optimized Scope‐Holder for Flexible Endoscopy (With Video)

**DOI:** 10.1111/den.70209

**Published:** 2026-06-30

**Authors:** Shinnosuke Nagano, Kota Momose, Yuji Ishii, Shuhei Yamaguchi, Shigeto Nakai, Takaomi Hagi, Kotaro Yamashita, Takuro Saito, Koji Tanaka, Tomoki Makino, Tsuyoshi Takahashi, Yukinori Kurokawa, Hidetoshi Eguchi, Yuichiro Doki, Kiyokazu Nakajima

**Affiliations:** ^1^ Department of Next Generation Endoscopic Intervention (Project ENGINE) The University of Osaka Graduate School of Medicine Osaka Japan; ^2^ Department of Gastroenterological Surgery The University of Osaka Graduate School of Medicine Osaka Japan; ^3^ Castem Co., Ltd Hiroshima Japan

**Keywords:** endoscopy‐related injuries, ergonomics, flexible endoscopy, scope‐holder

## Abstract

**Objectives:**

Growing endoscopy demand has increased physical workload and injury risk among endoscopists, while current ergonomic solutions remain limited. We developed a newly motion‐optimized endoscope‐holding device (scope‐holder) designed to reduce muscular burden, allowing each operator to adjust the scope position according to individual preference. This study assessed its ergonomic impacts in a simulated setting.

**Methods:**

Clinical endoscopists and nonmedical participants without any medical background were recruited to collect data. Each participant performed a simulated endoscopic marking task with and without the scope‐holder. We compared the procedure time of the task, evaluated muscle efforts in the upper arm, forearm, and shoulder using wireless electromyography, as well as mental workload using NASA‐TLX across the two settings.

**Results:**

A total of 26 participants, including 17 clinical endoscopists and 9 nonmedical participants, were enrolled. In the overall cohort, the scope‐holder assisted group significantly reduced activation of biceps brachii (*p* < 0.001), trapezius (*p* < 0.001), flexor carpi ulnaris (*p* = 0.049) muscles and NASA‐TLX scores (*p* = 0.003). Additionally, the assisted group achieved a shorter procedure time (*p* = 0.018) compared with the non‐assisted group. Subgroup analysis showed reduced muscle load in both clinical endoscopists and nonmedical participants (*p* < 0.05), with additional improvements in mental workload (*p* = 0.004) and the procedure time (*p* = 0.027) observed only in nonmedical participants, whereas clinical endoscopists showed no significant difference in mental workload (*p* = 0.299) and procedure time (*p* = 0.229).

**Conclusions:**

Our newly developed scope‐holder, designed to accommodate the operator's natural scope motion, effectively reduced physical and mental workload. It offers a practical solution to improve ergonomics in endoscopic clinical practice.

## Graphical abstract

An ergonomically optimized scope‐holder for flexible endoscopy was developed based on endoscopists’ left‐hand motion‐capture data to provide stable scope support while preserving the natural range of motion. In a simulated endoscopic marking task comparing non‐assisted and assisted conditions in 26 participants, scope‐holder assistance reduced muscle activation and NASA‐TLX scores, with shorter overall procedure time. These findings suggest that the device may reduce operator workload and improve ergonomics in flexible endoscopy.

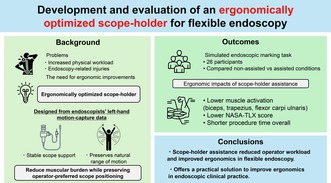

## 1 Introduction

The expansion of cancer screening programs and the widespread adoption of endoscopic treatment have markedly increased the physical and mental workload of endoscopists [1, 2, 3]. Consequently, the incidence of endoscopy‐related injuries (ERIs) among gastroenterologists has been rising [4, 5]. These injuries may disrupt daily activities, limit procedural performance due to pain or discomfort, and even lead to a loss of skilled professionals. Ultimately, such ergonomic issues may adversely affect patient care, highlighting the urgent need for ergonomic interventions in endoscopic practice.

Previously, we analyzed left‐hand movements and muscle activity during diagnostic and therapeutic upper gastrointestinal endoscopy, demonstrating that current endoscopic techniques, especially therapeutic ones, are not ergonomically optimized [6]. These findings emphasized the need for ergonomic improvements, such as workstation optimization, handle redesign, and supportive devices. Although the American Society for Gastrointestinal Endoscopy (ASGE) ergonomic guidelines recommend strategies like proper posture and monitor positioning [7, 8], few devices have been specifically designed for ergonomic benefit. Therefore, we developed a novel endoscope‐holding device (scope‐holder) that can be easily applied in clinical settings without modifying the endoscope itself, aiming to reduce endoscopists' workload.

Although several endoscope‐holding systems, such as belt‐like holders and patient bed–mounted antigravity support devices, have been reported previously [9, 10, 11], these systems typically secure the endoscope to the operator's waist or the patient's bed. Such rigid fixation may restrict instrument mobility and limit applicability to therapeutic procedures. In contrast, our device provides stable support for the endoscope body while maintaining an optimized range of motion for the operator's left hand, based on previously analyzed kinematic data of endoscopists. This design was intended to reduce left‐hand muscular load by supporting the endoscope body, while allowing each operator to adjust the scope position according to individual preference and preserving the fine control required for precise endoscopic manipulation. We hypothesized that these design features would reduce the physical and mental workload of endoscopists ergonomically. The present study aimed to evaluate the effectiveness of this device in terms of physical muscle activity, mental workload, and endoscopic performance.

## 2 Methods

### 2.1 Participants

We recruited clinical endoscopists and nonmedical participants without medical backgrounds. The nonmedical participants had never performed any endoscopic operation or training. Clinical endoscopists were classified as experts or novices; experts had performed more than 100 ESD procedures and were board‐certified by the Japan Gastroenterological Endoscopy Society, whereas novices did not meet these criteria.

### 2.2 Endoscope‐Holding Device

**FIGURE 1:**
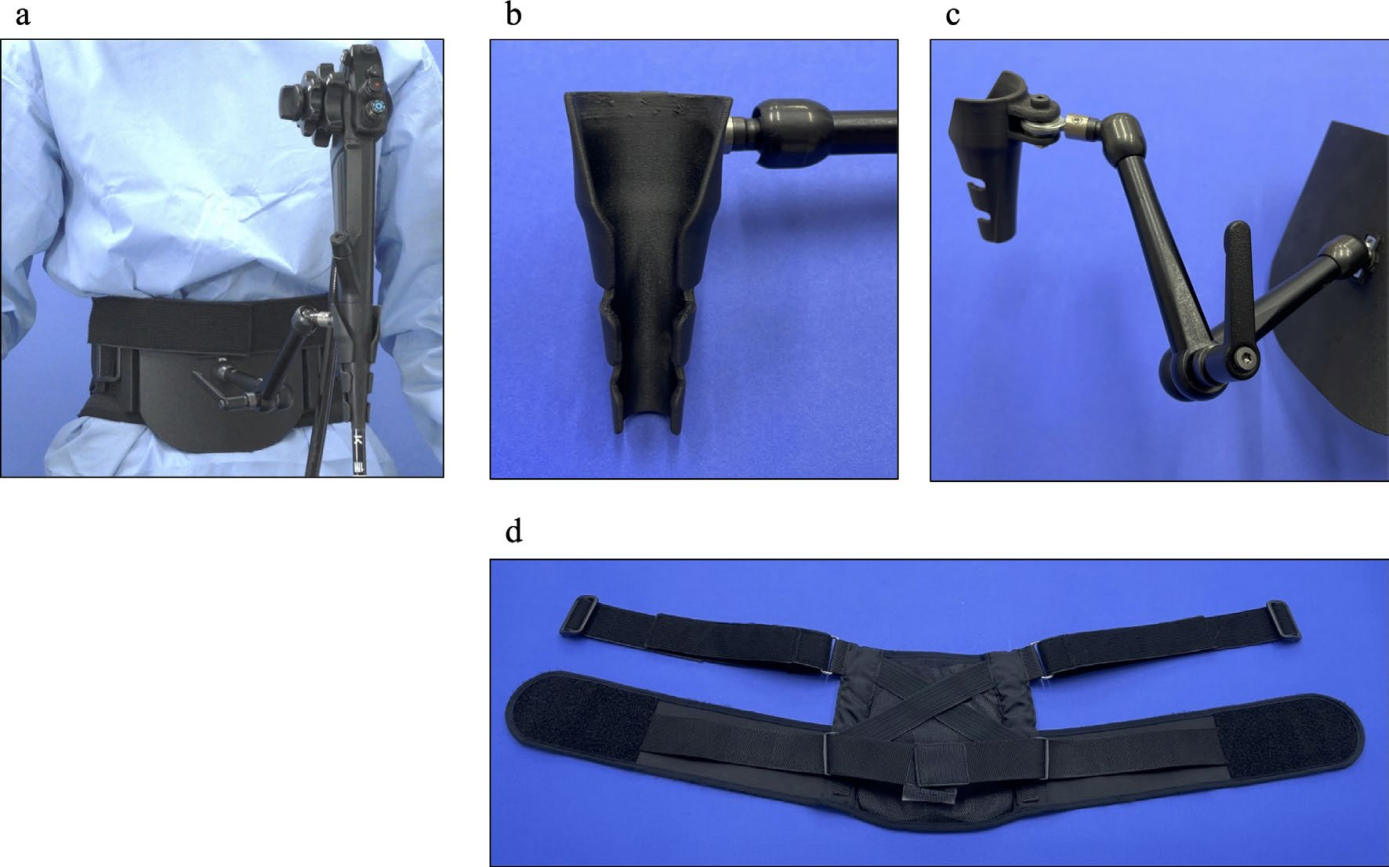
Overview of the endoscope‐holding device. Overall view of the endoscope‐holding device (a). The device consists of three main components: The holding unit (b), the articulated motion unit (c), and the belt fixation unit (d).

**FIGURE 2:**
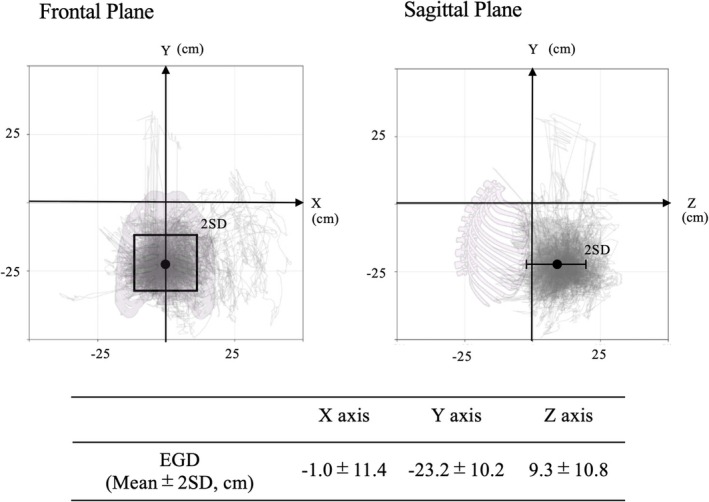
Movement range of the endoscopist's left hand holding the endoscope. The movement trajectories of the endoscope body during 25 endoscopic procedures were recorded using an optical motion capture system and plotted as light gray dots. The trajectories were projected onto the XY plane (frontal plane) and YZ plane (sagittal plane). The pink human skeleton represents the operator's body orientation in each plane. The large black dots indicate the mean coordinates of all plotted data points, and the ranges of ±2 SD are shown.

The scope‐holder is shown in Figure 1a and consists of three components: the holding unit, articulated motion unit, and belt fixation unit (Figure 1b–d). The holding unit allows 360° axial rotation of the endoscope while maintaining stable support (Figure 1b). The articulated motion unit was optimized based on previously analyzed left‐hand motion trajectories of endoscopists obtained using an optical motion capture system (OptiTrack Flex13; NaturalPoint, OR) (Figure 1c) [6]. Detailed motion tracking results are described in Supporting Material. It was designed to allow unrestricted movement within the observed mean ± 2SD ranges along all three axes (Figure 2), and was engineered with three rotational joints, enabling free three‐dimensional positioning and adjustable angulation (Video S1). A single locking lever enables rapid transition between fixation and repositioning. The belt fixation unit, designed as a dual waist belt, secures the device to the operator's torso and redistributes the weight of the endoscope from the upper extremity to the torso (Figure 1d). A demonstration of endoscopic procedures using the device is shown in Video S2.

### 2.3 Endoscopic Marking Task Model

We used the endoscopic marking task based on Tasks 3 and 5 from the fundamentals of endoscopic surgery program using an ex vivo ESD training model (Training model II, LM‐083; Olympus, Tokyo, Japan) [12, 13]. Participants performed an endoscopic marking task using a standard flexible endoscope (GIF‐Q260J; Olympus, Tokyo, Japan). Sequential markings were made on the mucosal surface within a double‐circled target area, numbered from 1 to 12, using a 1.5‐mm DualKnife J (KD‐655Q; Olympus, Tokyo, Japan) in swift coagulation mode. When a marking was placed outside the designated target area, the participant was instructed by an independent observer to repeat the marking at the same position. The task began with the first marking, and an independent investigator evaluated whether each marking remained within the double‐circled target and recorded the total procedural time. The endoscopy room was precisely arranged according to the American Society for Gastrointestinal Endoscopy (ASGE) ergonomic guidelines [7, 8].

### 2.4 Endoscopic Marking Procedures Without and With the Endoscope‐Holding Device

Each participant performed the endoscopic marking task under two conditions: without and with the scope‐holder. In the non‐assisted group, participants held the endoscope body with their left hand and operated it as in routine clinical practice. In the assisted group, participants mounted the scope‐holder, adjusted the articulating arm to a preferred position to stabilize the endoscope, and placed the endoscope body in the holder unit. Throughout the task, participants maintained the endoscope within the holder, which allowed them to keep their left hand, traditionally used to hold the scope, completely free. Participants were allowed to operate the endoscope using two approaches: they could either hold the endoscope body with the left hand and manipulate the control dials to steer the distal tip, as in the conventional technique, while the scope body was supported by the holding device, or they could rely entirely on the scope‐holder and keep the left hand detached from the endoscope, focusing solely on dial manipulation. Participants were not restricted to a single approach and were allowed to freely alternate between these two modes during the task.

A 60‐min instructional session was conducted simultaneously for all nonmedical participants (Supporting Material). Before the main task, all participants were allowed to practice for 10 min under both conditions. The order of experimental conditions was randomized for each participant to reduce potential learning and order effects, with a 5‐min rest between tasks.

### 2.5 Muscle Activation and Effort

Objective muscle activation was measured using wireless surface EMG (BioLog DL‐5500, DL‐510A; S&ME, Tokyo, Japan) [6]. EMG sensors were attached to three muscles during the marking task: left biceps brachii, left flexor carpi ulnaris, and left trapezius [6]. Detailed rationale for muscle selection and EMG recording conditions are provided in the Supporting Material. The root mean square (RMS) values were calculated from the EMG amplitudes within each analysis window [14]. Because muscle activity was compared within the same participants under two conditions, paired comparisons of RMS values were considered appropriate [15].

### 2.6 NASA‐TLX

After completing the task under each experimental condition, participants were asked to fill out the National Aeronautics and Space Administration Task Load Index (NASA‐TLX), a validated subjective measure of perceived workload [16, 17]. Details of the NASA‐TLX are provided in the Supporting Material.

### 2.7 Statistical Analysis

Statistical analyses were performed using the JMP software (version 17.0.0; SAS Institute, Cary, NC, USA). The comparison of the muscle activation, NASA‐TLX score and procedure time of the endoscopic marking task between scope‐holder non‐assisted and assisted group was analyzed using the Wilcoxon signed‐rank test. A *p* value < 0.05 was considered statistically significant.

## 3 Results

### 3.1 Participants

A total of 26 participants were enrolled in this study. There were 21 men and 5 females, including 17 clinical endoscopists and nine nonmedical participants. Their median (range) age was 36 (26–55) years, height was 170 (153–185) cm, and weight was 63.5 (45–98) kg. Baseline characteristics by subgroup are summarized in Table 1.

**TABLE 1:** The characteristics of participants.

		Clinical endoscopists (*n* = 17)	Nonmedical participants (*n* = 9)
Age (years)	Median (range)	35 (29–55)	40 (26–50)
Sex	Male/Female	13/4	8/1
Height (cm)	Median (range)	169 (153–185)	171 (164–178)
Weight (kg)	Median (range)	63 (45–80)	75 (58–98)
Experience	Expert/Novice	7/10	—

### 3.2 Muscle Activation and Effort

**FIGURE 3:**
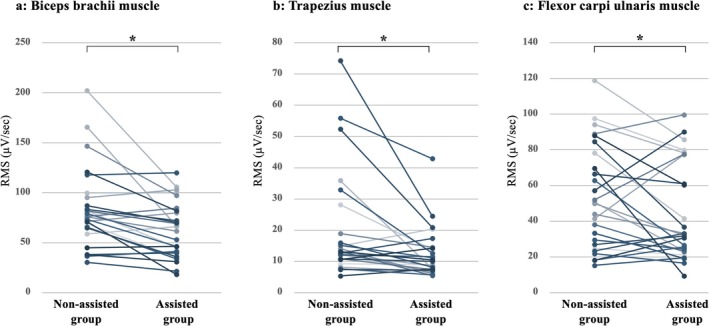
Comparison of muscle activation between the scope‐holder non‐assisted and assisted groups. Muscle activity of the biceps brachii (a), trapezius (b), and flexor carpi ulnaris (c) muscles for all 26 participants, expressed as root mean square (RMS) values. *p < 0.05.

The muscle activation of the three recorded muscles between the scope‐holder non‐assisted and assisted groups for all 26 participants is shown in Figure 3 and Table S1a. The assisted group showed significantly reduced muscle activity in the biceps brachii (*p* < 0.001), trapezius (*p* < 0.001), and flexor carpi ulnaris muscle (*p* = 0.049).

**FIGURE 4:**
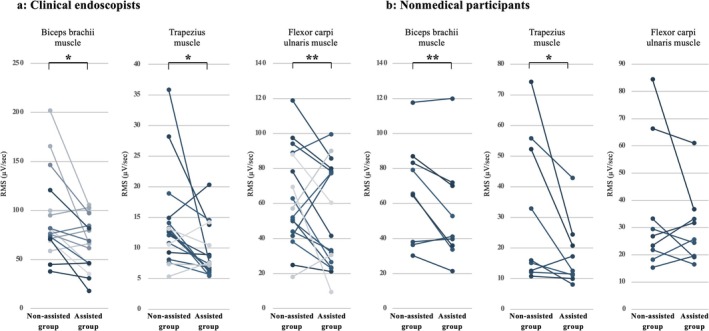
Subgroup analyses of muscle activation according to participant background. Comparisons of muscle activity between the scope‐holder non‐assisted and assisted groups in clinical endoscopists (a) and nonmedical participants (b). Muscle activation of the biceps brachii, trapezius, and flexor carpi ulnaris muscles is expressed as root mean square (RMS) values. *p < 0.05, **p < 0.01.

In subgroup analyses (Figure 4 and Table S1b), both clinical endoscopists and nonmedical participants showed significantly lower muscle activation in the trapezius muscle under the assisted condition compared with the non‐assisted condition (clinical endoscopists, *p* = 0.013; nonmedical participants, *p* = 0.027). In addition, a significant reduction in the biceps brachii was observed in clinical endoscopists (*p* = 0.013), whereas a similar trend was noted in nonmedical participants (*p* = 0.055). In contrast, no significant differences were observed in the flexor carpi ulnaris muscle between the two conditions in either group (clinical endoscopists: *p* = 0.098; nonmedical participants: *p* = 0.496). Furthermore, in a subgroup analysis within clinical endoscopists, no significant differences were observed in any of the three muscles in the expert group, whereas the novice group showed significant reductions in muscle activity in the biceps brachii (*p* = 0.027) and trapezius (*p* = 0.027) (Figure S1).

### 3.3 NASA‐TLX Score

**FIGURE 5:**
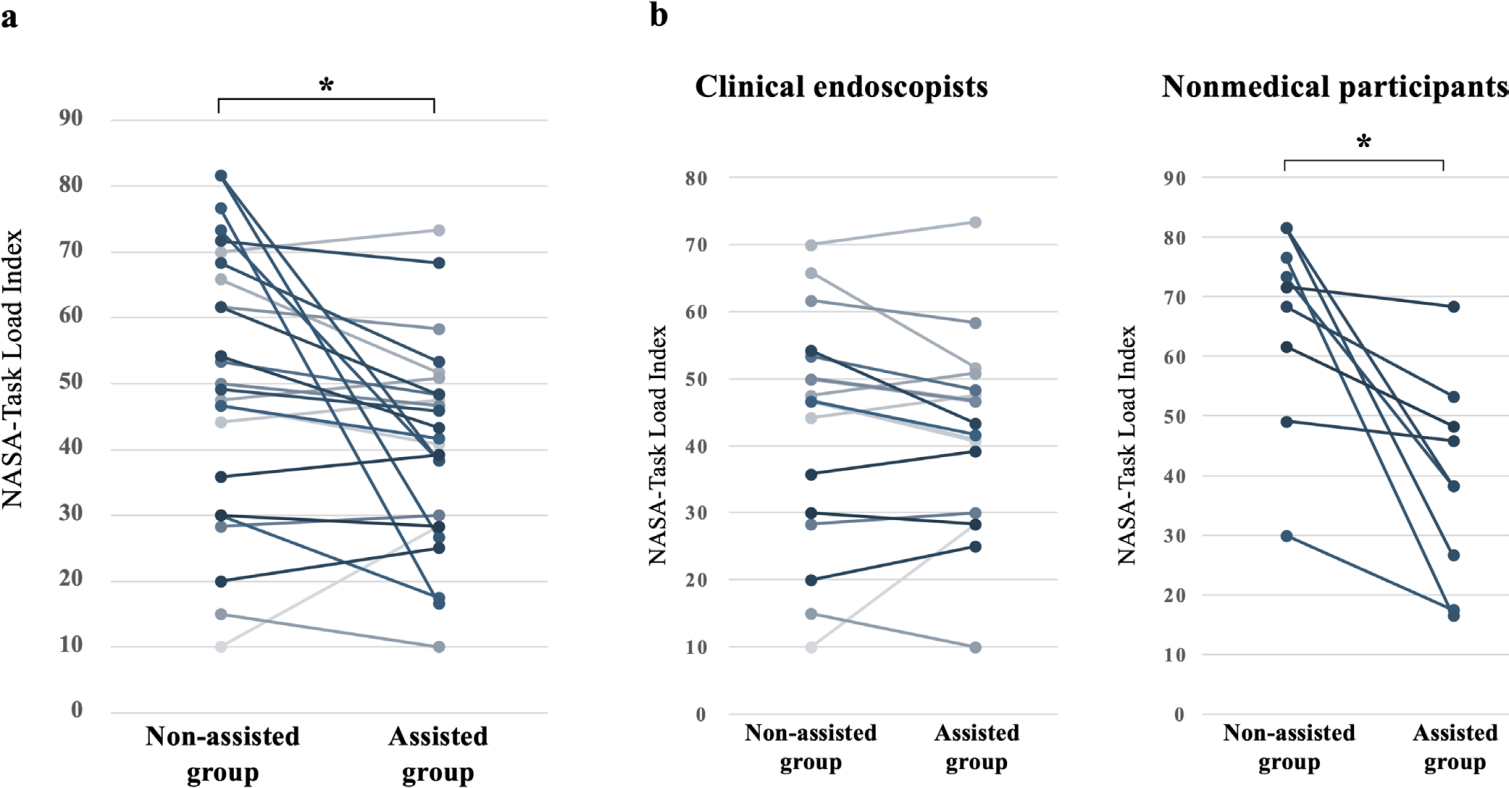
Comparison of mental workload assessed by NASA Task Load Index (NASA‐TLX). NASA‐TLX scores between the scope‐holder non‐assisted and assisted groups for all 26 participants (a) and subgroup analyses according to participant background (b). *p < 0.05.

The NASA‐TLX scores between the non‐assisted and assisted groups for all 26 participants are shown in Figure 5a. The median (range) score was 50.0 (10–81.7) in the non‐assisted group and 42.5 (10–73.3) in the assisted group. Overall, the NASA‐TLX score was significantly lower in the assisted group compared with the non‐assisted group (*p* = 0.003).

In a subgroup analysis (Figure 5b), among clinical endoscopists, no significant difference in the NASA‐TLX score was observed between the non‐assisted and assisted groups (*p* = 0.299). In contrast, among nonmedical participants, the score was significantly lower in the assisted group than in the non‐assisted group (*p* = 0.004). In subgroup analysis of clinical endoscopists, no significant difference in NASA‐TLX scores was observed in the expert group (*p* = 0.688), whereas a trend toward reduced scores was noted in the novice group (*p* = 0.072) (Figure S2).

### 3.4 The Procedure Time of the Endoscopic Marking Task

**FIGURE 6:**
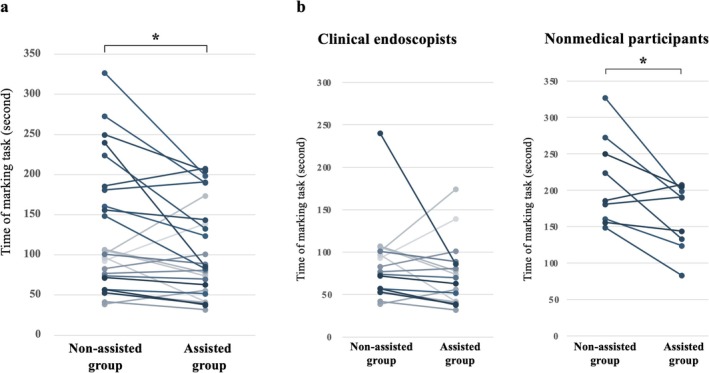
Comparison of procedure time for the endoscopic marking task. Procedure time between the scope‐holder non‐assisted and assisted groups for all 26 participants (a) and subgroup analyses according to participant background (b). *p < 0.05.

The procedure time of the endoscopic marking task between non‐assisted and assisted groups for all 26 participants is shown in Figure 6a. The median (range) procedure time was 101 (39–327) seconds in the non‐assisted group and 84.5 (32–208) seconds in the assisted group. The procedure time was significantly shorter in the assisted group compared with the non‐assisted group (*p* = 0.018).

In subgroup analyses (Figure 6b), among clinical endoscopists, no significant difference in procedure time was observed between the non‐assisted and assisted groups (*p* = 0.229). In contrast, among nonmedical participants, the procedure time was significantly shorter in the assisted group than in the non‐assisted group (*p* = 0.027). In a subgroup analysis of clinical endoscopists, no significant difference in procedure time was observed in either the expert group (*p* = 0.813) or the novice group (*p* = 0.225) (Figure S3).

## 4 Discussion

Our previous study demonstrated that current upper gastrointestinal endoscopic techniques, particularly therapeutic procedures, are not ergonomically optimized [6]. Although the ASGE guidelines recommend ergonomic strategies to reduce ERIs [7, 8], these measures remain insufficient in addressing operator workload. From an ergonomic standpoint, the ultimate solution may be a robot‐assisted endoscopic system [18, 19, 20]. However, the widespread implementation of flexible endoscope robotic systems remains limited due to technological complexity, procedural volume, and cost. Therefore, we sought to develop an endoscope‐holding device that could reduce operator strain without requiring major modifications to the conventional endoscope. Previous efforts have explored similar concepts [9, 10, 11], but their rigid fixation around the patient's bed and the endoscopist's waist may restrict scope maneuverability and limit adaptability to individual handling styles. In contrast, our scope‐holder was designed based on previously analyzed kinematic data of endoscopists' left‐hand movements [6], allowing sufficient range of motion for scope manipulation while providing stable support. This ergonomic design was conceived to accommodate individual variations in endoscope handling among operators, allowing for a reduction in muscle load while maintaining procedural freedom.

In the present ex vivo task evaluation, our new scope‐holder demonstrated clear ergonomic benefits, including reductions in muscle load and mental workload. EMG analysis is widely used to assess muscle load [21, 22, 23], and muscle activation levels are used to evaluate musculoskeletal disorder risk [24]. Therefore, reduced muscle activity is generally considered to reflect decreased muscular load and may contribute to injury prevention. In this study, the reduction in muscle activation was more pronounced in the biceps brachii and trapezius muscles than in the flexor carpi ulnaris, although a smaller but statistically significant reduction was also observed in the flexor carpi ulnaris. This finding may reflect the functional role of the flexor carpi ulnaris in wrist movements required for fine endoscopic manipulations, such as dial operation and torque control. Because these wrist movements remain essential even when using the scope‐holder, the observed reduction in muscle load in this muscle may have been relatively limited compared with the other muscles. Furthermore, procedure time was shorter in the overall cohort and among nonmedical participants, while no significant difference was observed among clinical endoscopists. These findings may suggest that optimizing the endoscope's range of motion allowed the device to reduce muscular activity in the operator's left hand without interfering with conventional endoscopic maneuvers. Although procedural accuracy was not assessed in this study, the shorter procedure time observed in some groups may reflect improved handling efficiency under the assisted condition.

With regard to clinical application, although the present task model simulated a static ESD procedure, the device's ergonomic advantages may be particularly relevant to various therapeutic endoscopy, where the range of motion is relatively limited but muscle load is high [6]. In clinical practice, larger scope movements may still be required, in which case the endoscope can be temporarily disengaged from the holder and manipulated conventionally. In contrast, the device may be more suitable for relatively static procedures, such as endoscopic retrograde cholangiopancreatography. However, patient movement, procedural complexity, and prolonged procedure time may affect device usability. Therefore, further refinement and clinical validation are warranted.

Subgroup analyses showed that our scope‐holder reduced muscle load in both clinical endoscopists and nonmedical participants. On the other hand, the assisted group demonstrated marked improvements in mental workload and procedural time among nonmedical participants, whereas no significant differences were observed among clinical endoscopists. Nonmedical participants with no prior endoscopic experience represent an ideal population for ergonomic evaluation, as they are not constrained by habitual techniques or pre‐existing assumptions regarding endoscope handling. Their relatively homogeneous baseline skill levels also allow clearer assessment of intrinsic device effects, consistent with established ergonomic approaches using task‐naïve individuals [11, 25]. In contrast, clinical endoscopists are accustomed to holding the endoscope and simultaneously manipulating the control dials with the left hand, which may introduce a bias and limit adaptation to a new device. The unfamiliar handling required by the holding device may account for the lack of improvement in subjective mental workload and procedure time in this group. Consistent with these findings, an exploratory analysis based on operator experience suggested that the ergonomic benefits of the device were more evident in novice than in expert endoscopists. The favorable procedural time observed in nonmedical participants suggests that the scope‐holder may be particularly useful for true beginners in endoscopy, supporting ergonomic handling and efficient task execution from the outset. From an ergonomic perspective, the present finding that endoscope stabilization reduced muscle activity suggests that, for clinical endoscopists accustomed to conventional techniques, achieving a truly ergonomic working environment may require not only new procedural adaptation but also further refinement of device design, potentially including advanced assistive or robotic systems [18, 19, 20].

This study has several limitations. First, it was conducted using an ex vivo model rather than in a real clinical setting and was designed as a pilot study with a limited sample size without a priori sample size calculation, which may limit the generalizability of the findings, particularly in the subgroup analyses. Second, the marking task represented a relatively simple procedure and did not encompass the full range of endoscopic maneuvers and, because each task was performed only once per condition, did not allow evaluation of operator fatigue during prolonged procedures. Third, incorrect markings were repeated and reflected in the total procedure time, but the number of incorrect attempts and procedural accuracy was not documented separately. Therefore, further studies will be needed to more directly assess procedural performance. Fourth, potential learning effects could not be completely excluded. Despite standardized training and randomization between two conditions, individual differences in skill acquisition and repeated task performance may have influenced the results. Finally, under the assisted condition, participants were allowed to alternate between two operating modes, but the extent to which each mode was used was not formally recorded. Therefore, mode‐specific effects on EMG activity could not be analyzed separately.

In conclusion, our newly developed endoscope‐holding device, designed to accommodate the operator's natural scope motion, effectively reduced physical and mental workload. It offers a practical solution to improve ergonomics in endoscopic clinical practice. Future studies should assess its impact during complex procedures and prolonged use, as well as its potential to prevent ERIs.

## Author Contributions

S.N., K.M., Y.I., S.Y., and K.N.: conception and design. S.N., K.M., Y.I., S.Y., and K.N.: analysis and interpretation of the data. S.N., Y.I., S.Y., and K.N.: drafting of the article. S.N., T.H., K.Y., T.S., K.T., T.M., T.T., and Y.K.: critical revision of the article for important intellectual content. H.E., Y.D., and K.N.: final approval of the article.

## Funding

This work was supported by the Ministry of Economy, Trade and Industry (METI), Japan, through the R&D Support Program for Growth‐oriented Technology SMEs (Grant Number JPJ005698).

## Ethics Statement

The authors have nothing to report.

## Conflicts of Interest

Y.I. and S.Y. are affiliated with Castem Co. Ltd., which is collaborating in the development of the endoscope‐holding device investigated in this study. In addition, the Department of Next Generation Endoscopic Intervention (Project ENGINE), Osaka University Graduate School of Medicine, receives collaborative research funding from Castem Co. Ltd. The other authors declare no competing interests.

## Supporting information


**Figure S1:** Subgroup analyses of muscle activation according to operator experience in clinical endoscopists. Comparisons of muscle activity between the scope‐holder non‐assisted and assisted groups in experts (a) and novices (b). Muscle activation of the biceps brachii, trapezius, and flexor carpi ulnaris muscles is expressed as root‐mean‐square (RMS) values. *p < 0.05.
**Figure S2:** Subgroup analyses of mental workload assessed by NASA Task Load Index (NASA‐TLX) according to operator experience in clinical endoscopists. Comparisons of NASA‐TLX scores between the scope‐holder non‐assisted and assisted groups in experts and novices. **p < 0.01.
**Figure S3:** Subgroup analyses of procedure time for the endoscopic marking task according to operator experience in clinical endoscopists. Comparisons of procedure time between the scope‐holder non‐assisted and assisted groups in experts and novices.

**Table S1:** Summary statistics for EMG outcomes under scope‐holder non‐assisted and assisted conditions.

**Data S1:** (a) Motion tracking results. Motion tracking was performed by defining the sternal manubrium as the origin, with the right‐to‐left body axis as the X‐axis, cranial‐to‐caudal axis as the Y‐axis, and anterior‐to‐posterior axis as the Z‐axis. The observed range of motion (mean ± 2SD) was −1.0 ± 11.4 cm on the X‐axis, −23.2 ± 10.2 cm on the Y‐axis, and 9.3 ± 10.8 cm on the Z‐axis. (b) Instructional session for nonmedical participants. A 60‐min instructional session was conducted simultaneously for all nonmedical participants. A single independent instructor explained the functions of the endoscope components, basic handling of the endoscope body and distal tip, suction and air/water operation, and the marking procedure using the DualKnife J. Participants then performed hands‐on practice to confirm familiarity with dial control and torque manipulation through rotation of the endoscope body. (c) Rationale for muscle selection and EMG recording conditions. EMG sensors were attached to three muscles during the marking task; left biceps brachii, left flexor carpi ulnaris, and left trapezius. These muscles were selected to represent key components of endoscopic manipulation. The biceps brachii reflects muscular load associated with scope holding and positional control. The flexor carpi ulnaris represents wrist movements required for dial manipulation and torque control. The trapezius reflects postural load related to upper limb and shoulder stabilization during the procedure. Resting EMG signals was recorded first. Raw EMG data were sampled at 1000 Hz and processed with a band‐pass filter ranging from 5 to 200 Hz, applying a −20 dB/decade roll‐off to minimize motion artifacts and high‐frequency noise. (d) Details of *NASA‐TLX*. National Aeronautics and Space Administration Task Load Index (NASA‐TLX) questionnaire assesses six dimensions—mental demand, physical demand, temporal demand, effort, performance, and frustration—and yields an overall workload score. Each subscale is rated on a 20‐point visual analogue scale, providing a composite measure of subjective mental and physical workload.

**Video S1:** Demonstration of scope‐holder mobility within the motion capture–derived range.

**Video S2:** Demonstration of the use of the endoscope‐holding device during endoscopic procedures.

## Data Availability

The data that support the findings of this study are available from the corresponding author upon reasonable request.
