# Supplementary figures and images for: Development and Evaluation of an Ergonomically Optimized Scope‐Holder for Flexible Endoscopy (With Video)
Source: Dig Endosc. 2026 Jun 30;38(7):e70209. doi: 10.1111/den.70209 (PMC13316967; doi:10.1111/den.70209)

**Fig. S1**

**a: Expert**

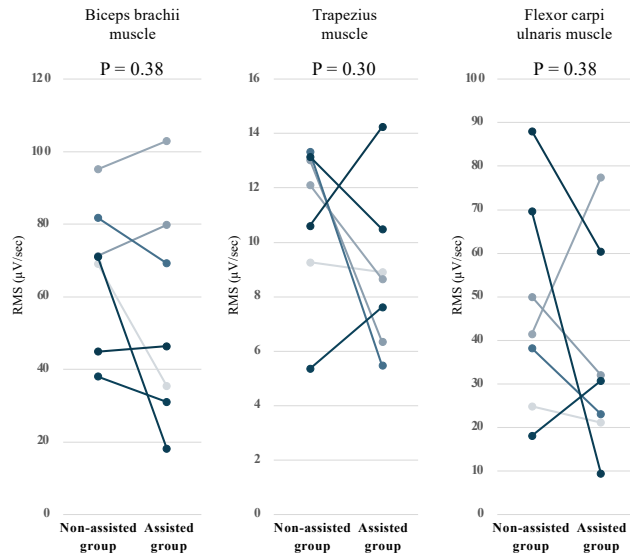

**b: Novice**

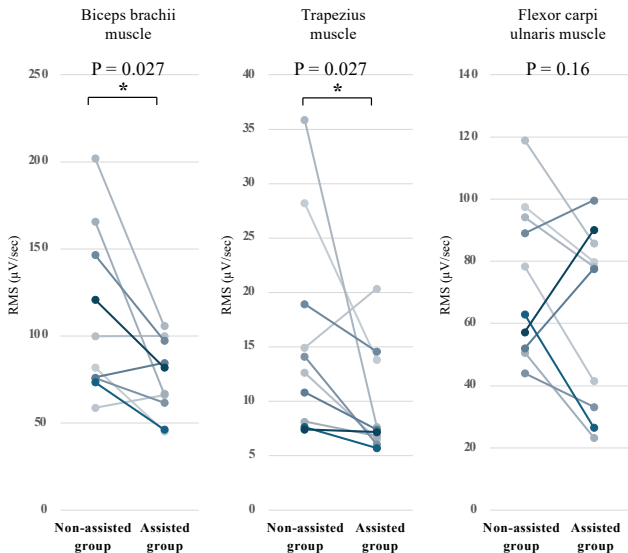

**Fig. S2**

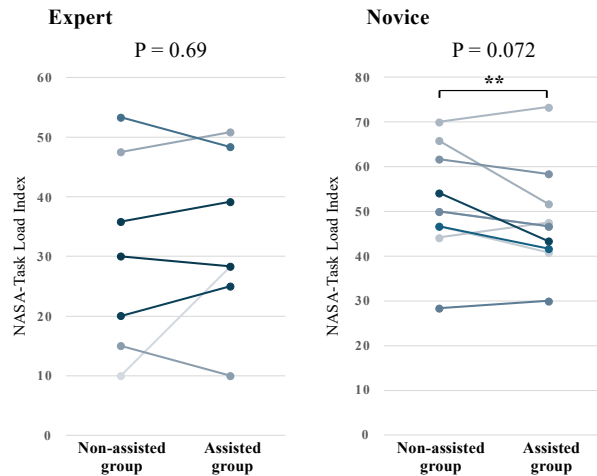

**Fig. S3**

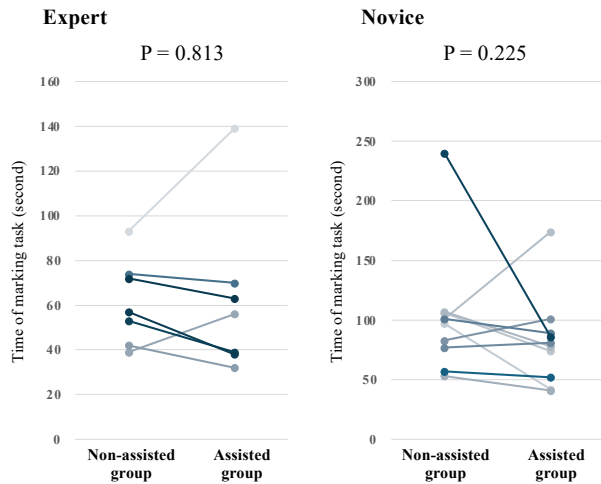

Supplement: Supplementary file 1 — Figure S1: Subgroup analyses of muscle activation according to operator experience in clinical endoscopists. Comparisons of muscle activity between the scope‐holder non‐assisted and assisted groups in experts (a) and novices (b). Muscle activation of the biceps brachii, trapezius, and flexor carpi ulnaris muscles is expressed as root‐mean‐square (RMS) values. *p < 0.05. Figure S2: Subgroup analyses of mental workload assessed by NASA Task Load Index (NASA‐TLX) according to operator experience in clinical endoscopists. Comparisons of NASA‐TLX scores between the scope‐holder non‐assisted and assisted groups in experts and novices. **p < 0.01. Figure S3: Subgroup analyses of procedure time for the endoscopic marking task according to operator experience in clinical endoscopists. Comparisons of procedure time between the scope‐holder non‐assisted and assisted groups in experts and novices. [file DEN-38-0-s003.pdf]
