# Supplementary material for: Development and Evaluation of an Ergonomically Optimized Scope‐Holder for Flexible Endoscopy (With Video)
Source: Dig Endosc. 2026 Jun 30;38(7):e70209. doi: 10.1111/den.70209 (PMC13316967; doi:10.1111/den.70209)
Supplement: Supplementary file 2 — Table S1: Summary statistics for EMG outcomes under scope‐holder non‐assisted and assisted conditions. [file DEN-38-0-s001.docx]

**Table S1. Summary statistics for EMG outcomes under scope-holder non-assisted and assisted conditions**

**a: Overall analysis**

|  | Non-assisted  RMS (µV/sec)  median (range) | Assisted  RMS (µV/sec)  median (range) | P |
| --- | --- | --- | --- |
| Biceps brachii muscle | 76.1 (30.3–202.0) | 63.9 (18.1–120.0) | <0.001 |
| Trapezius muscle | 13.1 (5.4–74.3) | 9.5 (5.5–42.9) | <0.001 |
| Flexor carpi ulnaris muscle | 50.4 (15.3–118.9) | 32.6 (9.4–99.6) | 0.049 |

**b: Subgroup analysis**

**Clinical endoscopists**

|  | Non-assisted  RMS (µV/sec)  median (range) | Assisted  RMS (µV/sec)  median (range) | P |
| --- | --- | --- | --- |
| Biceps brachii muscle | 76.3 (38.0–202.0) | 66.8 (18.1–105.7) | 0.013 |
| Trapezius muscle | 12.6 (5.4–35.9) | 7.6 (5.5–20.4) | 0.013 |
| Flexor carpi ulnaris muscle | 57.2 (18.1–118.9) | 41.6 (9.4–99.6) | 0.098 |

**Nonmedical participants**

|  | Non-assisted  RMS (µV/sec)  median (range) | Assisted  RMS (µV/sec)  median (range) | P |
| --- | --- | --- | --- |
| Biceps brachii muscle | 65.6 (30.3–117.7) | 41.2 (21.5–120.0) | 0.055 |
| Trapezius muscle | 16.0 (10.8–74.3) | 12.6 (8.2–42.9) | 0.027 |
| Flexor carpi ulnaris muscle | 26.9 (15.3–84.6) | 25.7 (16.6–61.0) | 0.496 |
