# Supplementary material for: Development and Evaluation of an Ergonomically Optimized Scope‐Holder for Flexible Endoscopy (With Video)
Source: Dig Endosc. 2026 Jun 30;38(7):e70209. doi: 10.1111/den.70209 (PMC13316967; doi:10.1111/den.70209)
Supplement: Supplementary file 3 — Data S1: (a) Motion tracking results. Motion tracking was performed by defining the sternal manubrium as the origin, with the right‐to‐left body axis as the X‐axis, cranial‐to‐caudal axis as the Y‐axis, and anterior‐to‐posterior axis as the Z‐axis. The observed range of motion (mean ± 2SD) was −1.0 ± 11.4 cm on the X‐axis, −23.2 ± 10.2 cm on the Y‐axis, and 9.3 ± 10.8 cm on the Z‐axis. (b) Instructional session for nonmedical participants. A 60‐min instructional session was conducted simultaneously for all nonmedical participants. A single independent instructor explained the functions of the endoscope components, basic handling of the endoscope body and distal tip, suction and air/water operation, and the marking procedure using the DualKnife J. Participants then performed hands‐on practice to confirm familiarity with dial control and torque manipulation through rotation of the endoscope body. (c) Rationale for muscle selection and EMG recording conditions. EMG sensors were attached to three muscles during the marking task; left biceps brachii, left flexor carpi ulnaris, and left trapezius. These muscles were selected to represent key components of endoscopic manipulation. The biceps brachii reflects muscular load associated with scope holding and positional control. The flexor carpi ulnaris represents wrist movements required for dial manipulation and torque control. The trapezius reflects postural load related to upper limb and shoulder stabilization during the procedure. Resting EMG signals was recorded first. Raw EMG data were sampled at 1000 Hz and processed with a band‐pass filter ranging from 5 to 200 Hz, applying a −20 dB/decade roll‐off to minimize motion artifacts and high‐frequency noise. (d) Details of NASA‐TLX. National Aeronautics and Space Administration Task Load Index (NASA‐TLX) questionnaire assesses six dimensions—mental demand, physical demand, temporal demand, effort, performance, and frustration—and yields an overall workload score. Each subsc [file DEN-38-0-s002.docx]

**Supplementary Document**

**(a) Motion tracking results**

Motion tracking was performed by defining the sternal manubrium as the origin, with the right-to-left body axis as the X-axis, cranial-to-caudal axis as the Y-axis, and anterior-to-posterior axis as the Z-axis. The observed range of motion (mean ± 2SD) was −1.0 ± 11.4 cm on the X-axis, −23.2 ± 10.2 cm on the Y-axis, and 9.3 ± 10.8 cm on the Z-axis.

**(b) Instructional session for nonmedical participants**

A 60-minute instructional session was conducted simultaneously for all nonmedical participants. A single independent instructor explained the functions of the endoscope components, basic handling of the endoscope body and distal tip, suction and air/water operation, and the marking procedure using the DualKnife J. Participants then performed hands-on practice to confirm familiarity with dial control and torque manipulation through rotation of the endoscope body.

**(c) Rationale for muscle selection and EMG recording conditions**

EMG sensors were attached to three muscles during the marking task; left biceps brachii, left flexor carpi ulnaris, and left trapezius. These muscles were selected to represent key components of endoscopic manipulation. The biceps brachii reflects muscular load associated with scope holding and positional control. The flexor carpi ulnaris represents wrist movements required for dial manipulation and torque control. The trapezius reflects postural load related to upper limb and shoulder stabilization during the procedure.

Resting EMG signals was recorded first. Raw EMG data were sampled at 1000 Hz and processed with a band-pass filter ranging from 5 to 200 Hz, applying a −20 dB/decade roll-off to minimize motion artifacts and high-frequency noise.

**(d)** **Details of *NASA-TLX***

National Aeronautics and Space Administration Task Load Index (NASA-TLX) questionnaire assesses six dimensions—mental demand, physical demand, temporal demand, effort, performance, and frustration—and yields an overall workload score. Each subscale is rated on a 20-point visual analogue scale, providing a composite measure of subjective mental and physical workload.
